# Transcriptional Regulation of Ovarian Steroidogenic Genes: Recent Findings Obtained from Stem Cell-Derived Steroidogenic Cells

**DOI:** 10.1155/2019/8973076

**Published:** 2019-04-01

**Authors:** Takashi Yazawa, Yoshitaka Imamichi, Toshio Sekiguchi, Kaoru Miyamoto, Junsuke Uwada, Md. Rafiqul Islam Khan, Nobuo Suzuki, Akihiro Umezawa, Takanobu Taniguchi

**Affiliations:** ^1^Department of Biochemistry, Asahikawa Medical University, Hokkaido 078-8510, Japan; ^2^Department of Pharmacology, Asahikawa Medical University, Hokkaido 078-8510, Japan; ^3^Noto Marine Laboratory, Division of Marine Environmental Studies, Institute of Nature and Environmental Technology, Kanazawa University, Ishikawa 927-0553, Japan; ^4^Department of Biochemistry, Faculty of Medical Sciences, University of Fukui, Fukui 910-1193, Japan; ^5^Department of Pharmacy, University of Rajshahi, Rajshahi, Bangladesh; ^6^National Research Institute for Child Health and Development, Tokyo 157-8535, Japan

## Abstract

Ovaries represent one of the primary steroidogenic organs, producing estrogen and progesterone under the regulation of gonadotropins during the estrous cycle. Gonadotropins fluctuate the expression of various steroidogenesis-related genes, such as those encoding steroidogenic enzymes, cholesterol deliverer, and electronic transporter. Steroidogenic factor-1 (SF-1)/adrenal 4-binding protein (Ad4BP)/NR5A1 and liver receptor homolog-1 (LRH-1) play important roles in these phenomena via transcriptional regulation. With the aid of cAMP, SF-1/Ad4BP and LRH-1 can induce the differentiation of stem cells into steroidogenic cells. This model is a useful tool for studying the molecular mechanisms of steroidogenesis. In this article, we will provide insight into the transcriptional regulation of steroidogenesis-related genes in ovaries that are revealed from stem cell-derived steroidogenic cells. Using the cells derived from the model, novel SF-1/Ad4BP- and LRH-1-regulated genes were identified by combined DNA microarray and promoter tiling array analyses. The interaction of SF-1/Ad4BP and LRH-1 with transcriptional regulators in the regulation of ovarian steroidogenesis was also revealed.

## 1. Introduction

**Figure 1:**
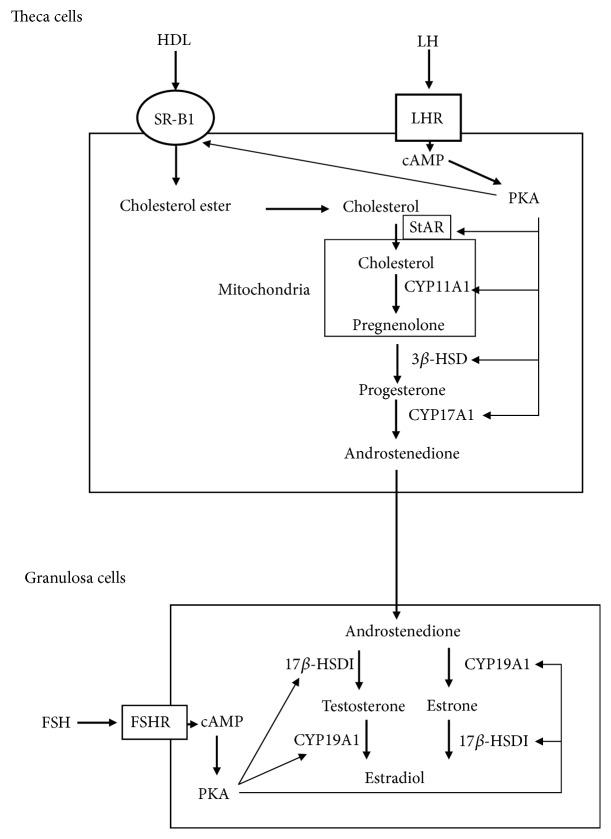
Schematic diagram of two-cell–two-gonadotropin theory in ovarian steroidogenesis. Theca cells autonomously produce androstenedione from cholesterol via the positive regulation of steroidogenic enzymes by LH/cAMP/PKA pathway. Granulosa cells convert androstenedione into estradiol. It is promoted by FSH/cAMP/PKA pathway.

In mammal, gonads and adrenal glands are primary organs that produce steroid hormones from cholesterol. Steroid hormones are involved in various physiological phenomena for the maintenance of homeostasis. Adrenal glucocorticoid and mineralocorticoid are essential for glucose metabolism, stress response, immunity, and fluid/electrolyte balance. Gonadal androgen and estrogen are important for sex differentiation and reproduction. These steroid hormones are produced from cholesterol through a series of reactions catalyzed by steroid cytochrome P450 (CYP) hydroxylases and hydroxysteroid dehydrogenases [1, 2]. The source of cholesterol for steroidogenesis primarily depends on cholesterol ester uptake from plasma proteins by lipoprotein receptors, such as scavenger receptor class B member 1 (SR-BI) [3, 4], although* de novo* synthesis and intracellular store also contribute to this process. Cholesterol transport from the outer to the inner mitochondria membrane by steroidogenic acute regulatory protein (StAR) represents a rate-limiting step of steroidogenesis [5]. Then, steroidogenesis begins with conversion of cholesterol into pregnenolone in mitochondria by the P450 side chain cleavage enzyme (P450scc/CYP11A1/Cyp11a1), an essential enzyme in the synthesis of all steroid hormones. Thereafter, various hormones are synthesized by tissue-specific CYP enzymes and hydroxysteroid dehydrogenases [1, 6, 7]. Earlier studies have demonstrated that ovaries secrete multiple steroid hormones such as pregnenolone, progesterone, 17*α*-progesterone, dehydroepiandrosterone, androstenedione, testosterone, estrone, and estradiol that depend on the estrous cycle [8]. Two types of somatic cells, follicular granulosa cells and surrounding theca cells, are responsible for ovarian steroidogenesis. Theca cells autonomously synthesize progesterone and androgen, whereas immature granulosa cells only convert theca cell-produced androgens into estrogens. These processes are finely regulated by two gonadotropins, follicle stimulating hormone (FSH) and luteinizing hormone (LH). During follicle development, LH stimulates the production of androgens in theca cells, which are converted to estrogens by FSH-inducible aromatase (CYP19A1/Cyp19a1) in granulosa cells (Figure 1). Such ovarian estrogen synthetic pathway represents the two-cell–two-gonadotropin theory [9, 10]. However, LH induces the differentiation of granulosa cells into autonomous progesterone-producing luteal cells during the ovulatory stage.

Transcriptional regulation of steroidogenesis-related genes, including those that encode steroidogenic enzymes, is an important step for regulating the aforementioned gonadotropin-dependent ovarian steroidogenesis. In general, gonadal steroidogenesis is activated by gonadotropin/cAMP/ cAMP-dependent protein kinase (PKA) pathway via induction of steroidogenic genes (Figure 1). These phenomena have been investigated using various* in vitro* culture systems, including follicle culture [11], primary cultures of theca and granulosa cells [12, 13], and established cell lines [14, 15]. Among them, granulosa cells collected from estrogen-primed immature rodents represent one of the most valuable models, as they can easily recapitulate the differentiation of nonsteroidogenic granulosa cells into steroidogenic luteal-like cells by FSH stimulation (even though LH is the physiological inducer of luteinization* in vivo*) [16, 17]. Human theca cells in primary and long-term culture are also valuable models for examining the theca cell differentiation, steroidogenesis, and pathology [12]. In addition to such current models, we have recently developed model systems to induce steroidogenic cells from nonsteroidogenic stem cells [18–22]. This model provides another useful tool to study the molecular mechanisms of steroidogenesis, since it can circumvent the limitation of cell number, lifespan, and stability of differentiation conditions [22, 23]. In this article, we will review the novel insights into transcriptional regulation of ovarian steroidogenesis-related genes that are obtained from this induction system, following the description of the roles of steroidogenic factor-1 (SF-1)/adrenal 4-binding protein (Ad4BP) in developing steroidogenic organs and differentiation of stem cells.

## 2. NR5A1/SF-1/Ad4BP: A Master Regulator of Organogenesis in Ovaries and Other Steroidogenic Organs

Although ovaries show different steroid hormone production profiles compared with testis and adrenal during adult life, they have a common developmental origin, a so-called adrenogonadal primordium (AGP) that mainly originates from the intermediate mesoderm and is localized on the coelomic epithelia of the developing urogenital ridge [24–26]. As embryonic development proceeds, AGP separates into two distinct populations, adrenocortical and gonadal primordia, characterized by the existence of chromaffin cell precursors and primordial germ cells (PGCs), respectively, which originate and migrate from other germ layers. SF-1/Ad4BP is one of the earliest markers of the appearance of AGP [24, 27]. Its expression is detectable within primitive urogenital ridges from the stage, when the AGP is not discernible based on morphological criteria (accession of PGCs) [27–29]. After separation into primitive gonads and adrenal, expression levels of SF-1/Ad4BP increased along with steroidogenesis initiation, although there are some species-specific differences in gonads. In rodent and pig, fetal testicular SF-1/Ad4BP is upregulated with the differentiation from bipotential gonads, whereas it is transiently downregulated in fetal ovaries until birth [28, 30, 31]: such sexual dimorphic gene expression reflects the difference in steroidogenic activity between fetal testes and ovaries. However, such sexually dimorphic expressions are never observed in humans [29] and other animals [32, 33]. SF-1/Ad4BP is important for steroidogenesis by regulating the transcription of various steroidogenesis-related genes. It belongs to the nuclear receptor (NR) superfamily and is very similar to liver receptor homolog-1 (LRH-1), as will be described in later sections. These factors constitute one of the NR subfamilies, officially termed as NR5A family proteins (SF-1/Ad4BP is NR5A1 and LRH-1 is NR5A2) [34]. Consistent with its role, ovarian SF-1/Ad4BP expression is detected in theca and granulosa cells, as well as in the corpus lutea to a lesser extent. In addition to steroidogenic enzymes, diverse groups of SF-1/Ad4BP target genes have been identified in ovaries and other steroidogenic organs [35–37].

Total* Nr5a1/*SF-1/Ad4BP-knockout (KO) mice die shortly after birth due to glucocorticoid deficiency and exhibit male-to-female sex reversal in external genitalia [35, 38, 39]. These phenotypes are caused by the complete loss of gonads and adrenals. Although the initial stages of gonadal and adrenal development occur in the absence of SF-1/Ad4BP, these organs regress and disappear during the following developmental stage, possibly due to the abnormality of glycolysis and pentose phosphate pathway [40]. This KO mice model demonstrated that SF-1/Ad4BP is crucial in determining steroidogenic organ fate* in vivo *and represents a master regulator for the development of these organs. A granulosa cell-specific KO (GCKO) model has shown that SF-1/Ad4BP plays important roles in steroidogenesis following the ovarian development [41]. In GCKO mice, the ovaries are hypoplastic with reduced oocytes and lack the corpora lutea [42]. Gonadotropin-induced steroid hormone production is also markedly reduced in this model. Thus, SF-1/Ad4BP is necessary not only for ovary development, but also for its physiological role throughout life.

## 3. Differentiation of MSCs into Steroidogenic Cells by SF-1/Ad4BP

In an early study, Milbrandt and colleagues attempted to induce steroidogenic cells from embryonic stem (ES) cells [43]. Ectopic expression of SF-1/Ad4BP was shown to direct differentiation of ES cells toward the steroidogenic lineage, and then Cyp11a1 mRNA was expressed after the addition of cAMP and retinoic acid (RA). However, these cells do not undergo* de novo* synthesis because supplementation of 20*α*-hydroxycholesterol, a membrane-permeable substrate, is necessary to produce progesterone, which is the only steroid produced by these cells. Major differences between these differentiated cells and natural steroidogenic cells are noted in cholesterol delivery and the steroidogenic pathway, including deficiencies of steroidogenic acute regulatory protein (StAR, a cholesterol delivery protein from the outer to the inner mitochondrial membrane in steroidogenic cells) and steroidogenic enzymes except for Cyp11a1 and 3*β*-HSD [18, 43, 44]. In addition, it is also very difficult to isolate clones expressing SF-1/Ad4BP from pluripotent cells such as ES cells and induced pluripotent stem cells [18, 21, 22], because SF-1/Ad4BP overexpression affects the survival and self-renewal of these pluripotent stem cells. These studies clearly indicate that SF-1/Ad4BP initiates the fate-determination program of the steroidogenic lineage in stem cells, even though it is not completed in pluripotent stem cells.

Based on this work, recent studies have focused on mesenchymal stem cells (MSCs) derived from bone marrow [18, 45]. MSCs are multipotent adult stem cells that differentiate into cells of mesodermal origin, such as adipocytes, chondrocytes, osteoblasts, and hematopoietic-supporting stroma both* in vivo* and* ex vivo* [46, 47]. Furthermore, MSCs are capable of generating cells of all three germ layers at least* in vitro.* Although MSCs were originally isolated from bone marrow (BM-MSCs) [48], they have also been derived from fat, placenta, umbilical cord blood and other tissues. Because MSCs are, like steroidogenic cells, of mesodermal origin, it was expected they are prone to execute their differentiation program.

**Figure 2:**
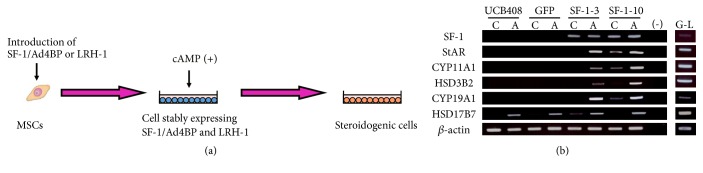
Differentiation of MSCs into steroidogenic cells. (a) Schematic diagram of induction of steroidogenic cells from MSCs by introduction of SF-1/Ad4BP or LRH, and cAMP-treatment. (b) Differentiation of UCB-MSCs into luteal-like cells by SF-1/Ad4BP. RT-PCR analysis of each gene in each cell with or without 8-bromo-cAMP for 2 d. Lanes G–L are granulosa-luteal cells from women undergoing oocyte retrieval for* in vitro* fertilization.

Indeed, MSCs have been completely differentiated into steroidogenic cells following stable expression of SF-1/Ad4BP and cAMP-treatment (Figure 2(a)) [18–22, 49]. While SF-1/Ad4BP induces morphological changes in murine MSCs, such as the accumulation of numerous lipid droplets, these cells hardly express steroidogenic enzymes or produce steroid hormones at detectable levels. However, SF-1/Ad4BP-expressing cells become markedly more positive for CYP11A1/Cyp11a1 after cAMP-treatment. These cells express many other steroidogenesis-related genes (SR-BI, StAR, 3*β*-HSD, and other P450 steroid hydroxylases) and autonomously produce steroid hormones including androgen, estrogen, progestin, glucocorticoid, and aldosterone. This approach differentiates human (h)BM-MSCs into high cortisol-producing cells in response to ACTH, which are very similar to fasciculata cells in the adrenal cortex. Adenovirus-mediated transient expression of SF-1/Ad4BP also differentiates BM-MSCs into steroidogenic cells with the capacity of* de novo* synthesis of various steroid hormones [45, 50–53]. In addition to BM-MSCs, various MSC types have been differentiated into steroidogenic cells* via* this method. For example, human umbilical cord blood- (hUCB-) derived MSCs are differentiated into progesterone-producing luteal-like cells (Figure 2(b)). However, as mentioned above, these methods are not applicable to pluripotent stem cells and embryonal carcinoma cells [18, 21, 45]. These results indicate that MSCs are suitable stem cells for the induction of steroidogenic cells. This hypothesis is supported by the fact that after predifferentiation into MSCs, ES cells can be subsequently differentiated into steroidogenic cells using SF-1/Ad4BP [21, 54]. It is also conclusive that SF-1/Ad4BP represents the master regulator of steroidogenesis. In fact, recent reports showed that SF-1/Ad4BP can reprogram some terminally differentiated cells, such as fibroblasts and endothelial cells [55].

## 4. Differentiation of MSCs into Steroidogenic Cells by Liver Receptor Homolog-1 (LRH-1) and Its Involvement in Luteal Steroidogenesis

The structural characteristics of SF-1/Ad4BP are very similar to liver receptor homolog-1 (LRH-1), which represents another NR5A family member and is designated as NR5A2. LRH-1 was originally identified in the liver and serves in various metabolic pathways, as well as cholesterol and bile acid homeostasis by regulating transcription of numerous genes [56–58]. In addition to the liver, LRH-1 is highly expressed in tissues of endodermal origin, such as the pancreas and intestine. It is also expressed in gonads, most abundantly in ovaries, and localized on granulosa cells and luteal cells [19, 20, 59–61]. LRH-1 shares various common characteristics with SF-1/Ad4BP, such as binding sequences, target genes, and cofactors, which can be applied to transcriptional regulation of steroidogenic genes. LRH-1 also activates the transcription of steroidogenesis-related genes [21, 62, 63].* Nr5a2*/LRH-1 KO mice exhibit abnormalities in steroidogenesis. Although total* Nr5a2/*LRH-1 KO embryos die around E6.5–7.5 days [64, 65], heterozygous and GCKO models revealed the importance of LRH-1 in steroidogenesis [55, 66–70]. In heterozygous* Nr5a2/*LRH-1-deficient male mice, testicular testosterone production is decreased along with the expression of steroidogenic enzymes and the development of sexual characteristics [71]. GCKO mice are infertile because of anovulation with impaired progesterone production [66]. These results strongly suggest that LRH-1 can also induce differentiation of MSCs into steroidogenic cells.

As for SF-1/Ad4BP, introduction of LRH-1 into BM-MSCs with the aid of cAMP induced expression of steroidogenic enzymes and differentiation into steroid hormone-producing cells [19, 21]. Expression of SF-1/Ad4BP was never induced in LRH-1-transduced cells, and vice versa. Therefore, LRH-1 could act as another master regulator for determining the fate of MSCs into the steroidogenic lineage. This phenomenon is likely to represent a situation where active progesterone production occurs in the ovarian corpus luteum, where LRH-1 is highly expressed, while SF-1/Ad4BP is expressed at very low levels [72]. Consistent with this hypothesis, Murphy and colleagues demonstrated using conditional KO mice models that* Nr5a2/*LRH-1 deletion in the corpus luteum reduced steroidogenesis, causing luteal insufficiency [67]. They also showed that depletion of* Nr5a2*/LRH-1 from granulosa cells impaired progesterone production and luteinization, even though SF-1/Ad4BP is expressed to some extent [66, 68]. Of note, intestinal glucocorticoid synthesis is also induced by LRH-1 [57, 73, 74]. When considered together, LRH-1 in addition to SF-1/Ad4BP can be another master regulator for steroidogenesis, as represented by luteal cells.

## 5. The Role of Transcriptional Coactivator PGC-1***α*** in Progesterone Production by Granulosa Cells

Steroidogenic properties of MSCs-derived cells vary markedly and depend on the tissues and species from which they are derived [18, 20, 52, 53, 72]. As mentioned above, BM-MSCs differentiated into cortisol-producing adrenocortical-like cells, and UCB-MSCs differentiated into granulosa-luteal-like cells, which produced high levels of progesterone [18, 20]. To determine why UCB-MSCs differentiate into luteal cells, gene expression profiles were compared with those of BM-MSCs using DNA microarray technology [20]. Among the identified genes, peroxisome proliferator-activated receptor *γ* coactivator-1*α* (PGC-1*α*) was expressed only in UCB-MSCs at relatively high levels. PGC-1*α* was originally discovered by Spiegelman and colleagues as coactivator of PPAR*γ* by conferring transcriptional regulation of its brown fat-specific target genes to PPAR*γ* [75]. It can also activate other NRs and transcription factors [76–78], as well as being an important regulator of metabolism and cell fate determination in a variety of tissues [77, 79–81].

**Figure 3:**
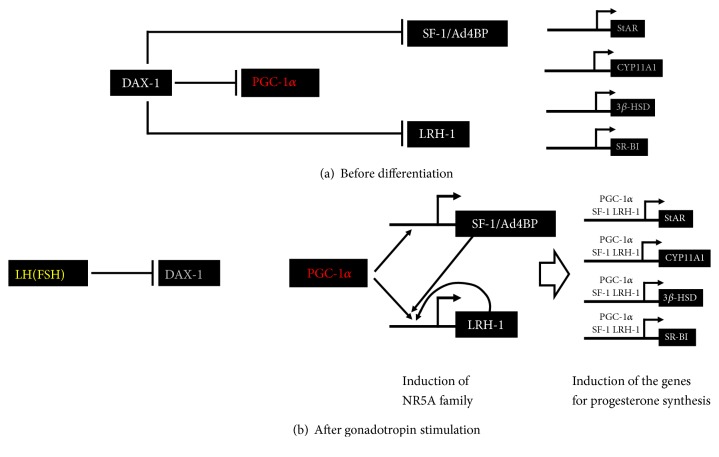
Regulation of granulosa cell differentiation by DAX-1 and PGC-1*α* via SF-1/Ad4BP and LRH-1. (a) During follicular development, the activities of SF-1/Ad4BP, LRH-1, and PGC-1*α* are repressed by DAX-1 in undifferentiated granulosa cells. Therefore, transcription of steroidogenic genes hardly occurred. (b) Gonadotropin induces the differentiation of granulosa cells into progesterone-producing cells. At this time, PGC-1*α* is released from DAX-1-mediated suppression via its reduction. This PGC-1*α* activation induces SF-1/Ad4BP and LRH-1. In particular, LRH-1 is highly induced by positive-feedback loop. Then, NR5A family proteins couple with PGC-1*α* to induce steroidogenic genes, such as StAR, CYP11A1, 3*β*-HSD, and SR-BI.

Consistent with the results in MSCs, ovarian PGC-1*α* was expressed in granulosa cells and colocalizes with SF-1/Ad4BP and LRH-1. PGC-1*α* acts as a very strong coactivator for SF-1/Ad4BP and LRH-1 via direct binding to their ligand-binding domains; its coactivation occurred in much higher levels compared to other coactivators (SRC-1, CBP, and P300) for SF-1/Ad4BP and LRH-1. Reporter assays revealed that PGC-1*α* activated the promoter activities of SF-1/Ad4BP and LRH-1 target genes, such as* StAR*,* CYP11A1*, and* HSD3B2*. Overexpression of PGC-1*α* induced expression of steroidogenic genes and progesterone production in both rat primary granulosa cells and human granulosa cell tumor–derived KGN cells. In addition to steroidogenic genes, PGC-1*α* also induced the expression of SF-1/Ad4BP and LRH-1. These results indicate that PGC-1*α* is involved in progesterone production in ovarian granulosa cells not only via potentiating transcriptional activities of the NR5A family proteins, but also via inducing their expression. Although PGC-1*α* induced the expression of both NR5A family genes, induction of LRH-1 occurred at a much higher level. As mentioned above, LRH-1 is highly expressed in the corpus luteum and SF-1/Ad4BP is expressed at very low levels [72]. Thus, PGC-1*α* may be a key factor promoting differentiation of granulosa cells into progesterone-producing luteal cells by acting on LRH-1 along multiple steps (Figure 3). Regarding the essential roles of LRH-1 in ovulation, recent reports suggest* PGC-1α* polymorphisms are associated with polycystic ovary syndrome (PCOS) [82]. PCOS is the most common endocrinopathy in women and the most common cause of anovulatory infertility, affecting 5%-10% of the population. It may be possible that dysfunction of PGC-1*α* in granulosa cells is one cause for anovulation in these patients.

## 6. Regulation of* NR0B1*/DAX-1 (Dosage Sensitive Sex Reversal, Adrenal Hypoplasia Congenital Critical Region on the X Chromosome, Gene 1) Expression by Gonadotropin

Nonsteroidogenic granulosa cells from preantral follicles differentiate into steroidogenic cells by FSH treatment under culture conditions, after which FSH rapidly induces various steroidogenic genes. However, the expression of SF-1/Ad4BP and LRH-1 is unaffected by FSH during earlier stages [83, 84]. In addition, PGC-1*α* mRNA and protein levels are relatively high even before FSH stimulation and are barely influenced by FSH [20]. This indicates that transactivation of SF-1/Ad4BP and LRH-1 via PGC-1*α* is repressed by other factor(s). Orphan nuclear receptor, DAX-1 (the encoded gene is officially termed* NR0B1*), is one of the most plausible candidates as a repressor. In ovaries, DAX-1 is localized on granulosa and theca cells [85]. The human* NR0B1* gene is located on the X chromosome at p21 and gives rise to 46,XY DSD/testicular dysgenesis through duplication of this region [86, 87].* NR0B1*/DAX-1 mutations are associated with pathogenesis of adrenal hypoplasia congenita and hypogonadotropic hypogonadism. DAX-1 represents an unusual nuclear receptor, as it lacks DNA-binding domain [88, 89]. Instead, the N-terminal domain contains three LXXLL-like motifs (NR boxes) that interact with the ligand-binding domain of NRs. DAX-1 represses the transcriptional activities of both SF-1/Ad4BP and LRH-1 via these NR boxes [20, 90]. Tissue localization of DAX-1 is very similar to SF-1/Ad4BP [85, 91], probably due to the fact that SF-1/Ad4BP regulates DAX-1 expression by direct binding to the promoter and enhancer regions at least during fetal life [85, 91, 92]. Therefore, ovarian DAX-1 is detectable in both granulosa and theca cells [85, 93].

In cultured granulosa cells, DAX-1 expression is acutely downregulated by FSH within 2h [20, 83]. This is consistent with the time for initiating induction of various SF-1/Ad4BP and LRH-1 target genes, including steroidogenic genes. Overexpression of DAX-1 inhibited promoter activities of SF-1/Ad4BP and LRH-1 target genes induced by FSH. In addition, DAX-1 suppressed not only the transactivation of NR5A proteins, but also PGC-1*α* dependent coactivation in a dose-dependent manner. LXXLL motifs of DAX-1 have much stronger affinities to SF-1/Ad4BP and LRH-1 than that of PGC-1*α* [20]. Therefore, a release from the DAX-1-mediated transcriptional repression could be an important mechanism for the transactivation of many SF-1/Ad4BP and LRH-1 target genes induced by gonadotropins (Figure 3). This should also be an important event during the differentiation of granulosa cells into steroidogenic luteal cells, because DAX-1 expression is further suppressed in luteal stage. Consistent with this hypothesis, Zeleznik and colleagues showed that overexpression of DAX-1 repressed the FSH-induced progesterone and estradiol production in granulosa cells [94]. Furthermore, LH and ACTH stimulate the steroidogenesis with a reduction of DAX-1 expression in theca, Leydig, and adrenocortical cells [95, 96]. Therefore, downregulation of DAX-1 expression is an important mechanism for promoting the steroidogenesis by pituitary hormones in primary steroidogenic organs.

## 7. Transcriptional Regulation of Electron Transporter That Transfers Electron to Steroidogenic Enzymes

To identify novel SF-1/Ad4BP and LRH-1 target genes, genome-wide analyses by a promoter tiling array (chip-on-chip) and DNA microarray were performed using MSCs-derived steroidogenic cells [97, 98]. In these studies, multiple genes were considered as possible targets of NR5A family proteins, including some electron transporter genes.

Steroidogenesis is catalyzed by various cytochrome P450 enzymes, which have to receive electrons from nicotinamide adenine dinucleotide (NADPH) through their redox partners for the catalytic reactions [99]. Therefore, the activities of P450 enzymes are determined not only by their own expression levels, but also by the rate of electron transfer from redox partners. It is then probable that expression of these redox genes is also regulated by pituitary hormones and NR5A family proteins, as in the case of P450 enzymes. We investigated this possibility using stem cell-derived steroidogenic cells.

**Figure 4:**
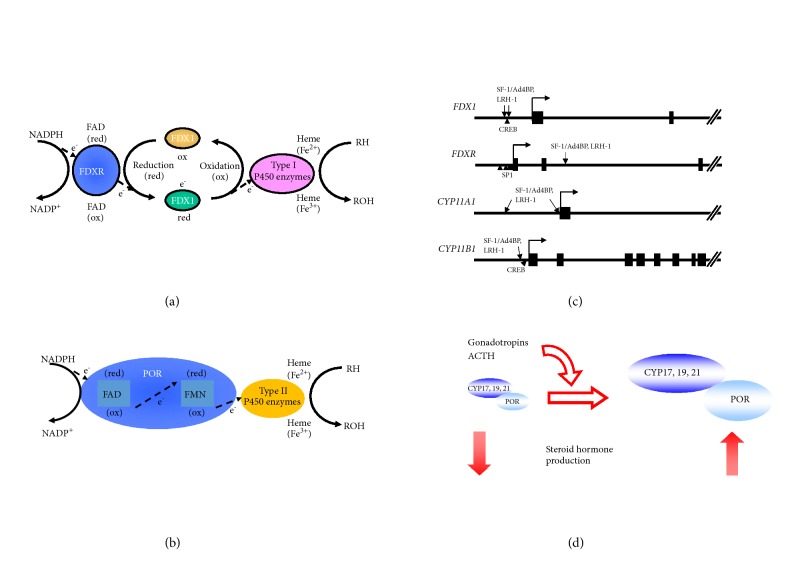
P450 enzymes and their redox partners in steroidogenesis. (a) Schematic diagram of electron transfer from NADPH to mitochondrial type I P450 enzymes by FDXR and FDX1. At first, NADPH passes the electrons to the nonreduced (ox) FAD moiety of FDXR. Then, reduced (red) FDXR passes them to FDX1, followed by the transfer to P450 enzymes and hydroxylation of cholesterol and its metabolites. (b) Schematic diagram of electron transfer from NADH to microsomal type II P450 enzymes by POR. POR receives the electrons from NADPH by using its FAD moiety, and red FAD passes them to its FMN. Then, electrons are transferred to P450 enzymes to activate the steroid hormone production. (c) The binding sites of NR5A family and other transcription factors, which are essential for the transcription of FDX, FDXR, and ovarian type I P450 genes. (d) Pituitary hormones promote the steroidogenesis by enhancing the transcription of both mitochondrial P450 enzymes and POR.

The human genome contains 57 P450 genes categorized into two classes based on their properties: type I and II enzymes. Each class occurs on different intracellular localizations and is coupled with different redox partners. Type I enzymes are localized on mitochondria and receive electrons from NADPH mediated by ferredoxin (FDX) and ferredoxin reductase (FDXR: Figure 4(a)). Conversely, type II enzymes are located in the endoplasmic reticulum and receive electrons from NADPH mediated by P450 oxidoreductase (POR) (Figure 4(b)). The results from genome-wide analyses suggest that SF-1/Ad4BP can control both pathways.

### 7.1. Transcriptional Regulation of FDX1 and FDXR by NR5A Family Proteins

Among type I P450 (CYP) enzymes, P450scc (CYP11A1) and CYP11B1 use FDX and FDXR as redox partners for receiving electron from NADPH. Although CYP11B1 is known to represent glucocorticoid-synthesizing enzyme in the adrenal gland, we have shown it is induced by LH/hCG pathway in ovarian theca cells and testicular Leydig cells [49, 100]. To pass electrons to these enzymes, NADPH initially binds to FDXR, flavoprotein localized on the mitochondrial inner membrane (Figure 4(a)) [99]. Using its flavin adenine dinucleotide (FAD) moiety, FDXR transfers electrons derived from NADPH to an iron sulfur (Fe_2_S_2_) cluster of FDX1. FDX1 then interacts with P450 enzymes and donates electrons to progress steroid hormone synthetic reactions. Chromatin immunoprecipitation (ChIP) analyses suggested that both FDX1 and FDXR genes are targets of SF-1/Ad4BP and LRH-1 [101, 102]. DNA microarray analyses also reveal that FDX1 is upregulated by SF-1/Ad4BP and cAMP during differentiation.

Consistent with the results of stem cell-derived steroidogenic cells, FDX1 is rapidly induced by FSH in cultured granulosa cells [101, 103, 104]. ChIP analyses using stem cell-derived cells revealed that the binding of SF-1/Ad4BP is enriched in the region of about 1kb upstream to transcription start site (TSS) of the* FDX1* gene. Reporter and gel mobility shift assays indicate that there are two SF-1/Ad4BP-binding sites within in this region, which are important for the* FDX1* promoter activity under 8Br-cAMP stimulation. LRH-1 can also bind to these sites and activate the transcription. In addition to NR5A family proteins, cAMP response element binding protein (CREB), which binds to CRE-like sequence adjacent to the both SF-1/Ad4BP-binding sites, was critical for the transcription of FDX1 by the stimulation of cAMP. Taken together, it is probable that the synergistic action of SF-1/Ad4BP and CREB is responsible for the acute induction of FDX1 gene by FSH/cAMP pathway in ovarian granulosa cells. Such regulation can be applicable to other FSH-inducible genes [83, 105–107].

In contrast to FDX1, the expression of FDXR is largely unaffected by FSH and cAMP in granulosa cells and stem cell–derived steroidogenic cells [102, 108]. FDXR is ubiquitously expressed in various tissues, despite being highly expressed in steroidogenic tissues including gonads and adrenal glands [108]. Therefore, it is reasonable that there are powerful basal elements near TSS; this region contains six elements closely resembling the consensus sequences for SP1 [109]. On the other hand, it was revealed by our ChIP analysis that SF-1/Ad4BP binds to the intronic region of* FDXR* gene [102, 110]. This region enhanced the activity of its own proximal promoter, containing SP1-binding sites. It was also shown that SF-1/Ad4BP- binding to this site induces dynamic changes of chromatin architectures. Introduction of SF-1/Ad4BP into MSCs clearly increased the enrichment of active chromatin marks, acetylated lysine 27 on histone 3 (H3K27ac) and dimethylated lysine 4 on histone 3 (H3K4me2) of intronic region of* FDXR*, compared with control cells. These data demonstrated that the intronic SF-1/Ad4BP binding region of FDXR functions as an enhancer to cause the higher expression of FDXR in primary steroidogenic organs including ovary.

Ovarian type I P450 enzymes are target genes of SF-1/Ad4BP and LRH-1 [105, 111, 112]. Thus, SF-1/Ad4BP and LRH-1 control the steroidogenesis by regulating not only the transcription of steroidogenic enzymes, but also the transcription of FDX and FDXR (Figure 4(c)).

### 7.2. Regulation of POR Expression by Gonadotropins

P450c17 (CYP17A1 and CYP19A1) represents ovarian type II enzymes, which use POR as redox partners for receiving electron from NADPH. POR is a membrane-bound flavoprotein, expressed ubiquitously with more or less variable expression levels among different tissues [99]. It contains not only FAD moiety, but also flavin mononucleotide (FMN), electron acceptor from FAD as Fe_2_S_2_ cluster of FDX1. Therefore, POR can transfer NADPH-derived electrons directly to P450 enzymes (Figure 4(b)) [99]. However, the microsomal P450 component is found in a great molar excess to POR, especially in steroidogenic tissues [113]. This is possible to cause a profound influence on steroidogenesis, when steroidogenic enzymes are acutely induced by pituitary hormones. This hypothesis might be supported by the fact that* POR* deficient patients exhibit skeletal dysplasia referred to as Antley-Bixler syndrome (ABS) that is accompanied with impaired steroidogenesis, resulting in adrenal dysfunction, disorders of sexual development, and maternal virilization during pregnancy [114–117].

DNA microarray analyses demonstrated that POR expression is increased with the differentiation of MSCs into steroidogenic cells by SF-1/Ad4BP and cAMP [97]. As in the case of MSCs-derived steroidogenic cells, POR was induced by FSH and hCG with CYP19A1 in rat granulosa cells. POR expression was also increased by LH in theca cells. In the transfection experiments, expression of POR enhanced the aromatase activity in dose-dependent manner, even though aromatase protein levels were constant. On the other hand, knockdown of endogenous POR proteins in KGN cells led to the reduction of estrogen production. These results indicate that POR is one of the gonadotropin-regulatable genes in ovarian granulosa cells, and this regulation should cause the augmentation of estrogen production by gonadotropins. It was also reported in adrenocortical cell lines that ACTH or cAMP increases the expression of POR [118, 119]. In addition, Hall and colleagues showed* in vitro* that the activity of purified CYP17A1 was increased by the addition of POR proteins in a dose-dependent manner, resulting in increased androgen production [120–122]. Therefore, it is conceivable that POR expression is regulated by the pituitary hormones with steroidogenic enzymes in steroidogenic cells (Figure 4(d)).

Although the mechanisms of transcriptional regulation by pituitary hormones remained unknown yet, Ogata and colleagues have analyzed the pivotal elements of the proximal promoter of human* POR* [123]. The* POR* gene spans more than 70 kb, including coding 1-15 exons and an untranslated exon 1 (exon 1U) residing about 38.8 kb upstream of the coding exon 1 [124]. The region around exon 1U was completely unmethylated CpG-rich region in various cells [123]. In addition, in silico analysis suggests that this region exhibits promoter-associated histone marks. Reporter, electron mobility shift assays and ChIP analyses demonstrated that SP1 proteins bind to at least three binding sites within this region and markedly activate the promoter activities. Promoter activities were undetectable in SP family-deficient cells. These results indicate that SP1-binding sites represent an essential element for the transcription of* POR* gene. Consistent with these experimental results, some ABS patients had a deletion of this region, and* POR* gene was never transcribed from this allele [123, 125].

## 8. Conclusion

We have shown that MSCs-derived steroidogenic cells provide an important tool for investigating the transcriptional regulation of ovarian steroidogenesis-related genes. Especially, it contributes to understand the function of SF-1/Ad4BP and LRH-1 by revealing the regulation of their transcriptional activity and their novel target genes. This system is also useful for studying other steroidogenic phenomena. Ovarian-specific LRH-1 isoform was also identified based on above studies [126, 127]. However, many mysteries remained in ovarian steroidogenesis, yet. Among them, the cause of the difference between granulosa and theca cells on steroidogenic properties is not clear, which is the essence of ovarian steroidogenesis, two-cell–two-gonadotropin theory. The difference of SF-1/Ad4BP and LRH-1 function in steroidogenesis is also unknown. Further studies will be necessary for the comprehensive elucidation of such mysteries.
